# Neprilysin Is Poorly Expressed in the Prefrontal Cortex of Aged Dogs with Cognitive Dysfunction Syndrome

**DOI:** 10.1155/2014/483281

**Published:** 2014-01-06

**Authors:** Jesús Canudas, Daniel Insua, Leticia Sarasa, Ángela González-Martínez, María Luisa Suárez, Germán Santamarina, Pedro Pesini, Manuel Sarasa

**Affiliations:** ^1^Araclon Biotech Ltd., Vía Hispanidad 21, 50009 Zaragoza, Spain; ^2^Departamento de Ciencias Clínicas Veterinarias, Facultad de Veterinaria de Lugo, Universidad de Santiago de Compostela, 27002 Lugo, Spain

## Abstract

Neprilysin (NEP) is the principal amyloid **β** (A**β**) degrading peptidase; this activity may protect against Alzheimer's disease (AD), the most important age-related neurodegenerative process. The aim of this work was to analyze NEP mRNA expression in the frontal cortex of dogs with and without canine cognitive dysfunction syndrome (CDS), which is considered a natural model for AD. Expression of canine cerebral NEP mRNA was assessed by RT-PCR followed by qPCR in young, aged-cognitively unimpaired (CU), and aged-cognitively impaired (CI) dogs. On average, aged-CI dogs showed 80% (*P* < 0.01) lower expression levels of NEP mRNA than their aged-CU counterparts. Furthermore, the standard deviation of the qPCR measurements was more than 6 times higher in the cognitively healthy animals (young and aged-CU) than in the aged-CI group. Another interesting find is the determination of a positive correlation between NEP expression and the number of cholinergic neurons in basal telencephalon, indicating a probable connection between both events in these types of neurodegeneration processes. These results suggest that high expression levels of NEP might be a protective factor for canine CDS and, most likely, for other A**β**-associated neurodegenerative diseases, such as AD.

## 1. Introduction

Amyloid *β* (A*β*) peptides are considered to be the main agents implicated in Alzheimer's disease (AD), the most common dementia [1]. Currently, researchers believe that an imbalance between production and clearance of A*β* peptides (especially A*β*_1–40_ and A*β*_1–42_) leads to the accumulation of A*β* peptides in the brain [2, 3]. The two most important mechanisms implicated in the elimination of brain A*β* are active transport through the blood brain barrier and the enzymatic degradation of A*β* peptides by peptidases, such as neprilysin (NEP), insulin-degrading enzyme (IDE), endothelin-converting enzymes (1 and 2), or plasmin. Of these, NEP is the most relevant protease in A*β* degradation regarding either its monomeric or oligomeric A*β* conformations [4, 5].

The gene that encodes NEP (also named MME) is expressed at the highest level in the kidney and at lower levels in other tissues including the lung, adrenal glands, intestine, and central nervous system [6]. In the brain, areas with higher A*β* accumulation (hippocampus, temporal, and frontal cortex) express lower levels of NEP than areas with lower A*β* deposition (cerebellum or caudate nucleus) [7, 8]. On the other hand, some studies have found a significant age-related downregulation of NEP protein in the mouse hippocampus [9, 10] and in human frontal and temporal cortex [11]. Nevertheless, in other studies, NEP protein levels did not correlate with age in human controls, NEP activity being significantly higher in elderly than in younger people [12]. Another significant investigation has shown that mice overexpressing both NEP and human APP, with the Swedish and Indiana mutations, in neurons had reduced brain A*β* burdens and presented less cognitive impairment [13]. All these findings suggest that NEP may play an important protective role against AD and could be an important therapeutic target [14].

The canine has been pointed as an appropriate model for the study of neurodegenerative diseases because dogs can, naturally, suffer from an age-related cognitive dysfunction syndrome (CDS), which reproduces core clinical and histopathological aspects of AD [15–21]. In addition, the dog's and human's enzymatic machineries for *β*-amyloid processing are highly homologous, with identical sequences for the A*β*_1–42_ peptide [22, 23].

In the present work, we have investigated the expression of NEP mRNA in the prefrontal cortex (gyrus proreus) of the dog, an early affected region in CDS and AD, to explore if there are differences in cortical NEP expression related to age or cognitive status.

## 2. Materials and Methods

### 2.1. Animals

Six young (<4 years) and 12 aged dogs (>11 years) were used in this study (Table 1). After systematic clinical examinations of their nervous systems, the aged dogs were separated into aged-cognitively impaired (CI) and unimpaired (CU) groups (6 animals per group) via questionnaires given to the owners [24]. Euthanasia was prescribed for all animals for humanitarian reasons, when their severe impairment was not compatible with a minimal quality of life. Brains were obtained with the owners' explicit consent. Immediately after brain extraction, a tissue block from the gyrus proreus was dissected and stored at −80°C until processing. All animals were treated according to the European and Spanish legislation on animal handling (86/609/EU, Real Decreto 1201/2005), and the experimental procedures were approved by the Ethical Committee of the University of Santiago.

### 2.2. RNA Extraction

Total RNA was isolated using the TRIzol reagent (Life technologies, Madrid, Spain) in accordance with the manufacturer's recommendations. The concentrations and purity of RNA were measured in a spectrophotometer at 260 and 280 nm. The RNA integrity was analyzed by RNA-agarose gel electrophoresis. RNA extractions were treated with DNase I (Life technologies, Madrid, Spain) to eliminate residual DNA contamination.

### 2.3. RT-PCR

To obtain cDNAs, 1.5 *μ*g of total RNA was processed for reverse transcription using the SuperScript First-Strand Synthesis System for RT-PCR (Life technologies, Madrid, Spain) according to the manufacturer's instructions. The NEP, IDE, and Ubiquitin (Ubi) cDNAs were amplified using respective primer pairs (see Table 2) in PCR reactions. The composition of the PCR reactions was as follows: 1x PCR reaction buffer, 0.02 U/*μ*L of platinum *Taq* DNA polymerase (Life technologies, Madrid, Spain), 1 *μ*L of cDNA, 1.5 mM MgCl_2_, 0.2 mM of each deoxynucleotide triphosphate, and 0.2 *μ*M of each primer. The amplification conditions included the following: an initial denaturation step for 2 minutes at 94°C; 34 amplification cycles with denaturation for 30 seconds at 94°C, 1 minute at an annealing temperature dependent on the pair of primers (see Table 2), and extension for 1 minute at 72°C; and a final extension for 10 minutes at 72°C. The reactions were conducted in a Veriti 96 Thermal Cycler (Applied Biosystems, Madrid, Spain). The PCR products were analyzed by electrophoresis in agarose gels stained with GelRed (Biotium, Hayward, CA) and visualized in a Gel Doc 1000 (Bio Rad, Madrid, Spain).

### 2.4. Quantitative PCR

To quantify the NEP cDNA of the prefrontal cortex of each dog, reverse transcription of total RNA was conducted to amplify the cDNAs of NEP and Ubi (our internal standard) using the Power SYBR Green RNA-to-C_T_ 1-Step Kit (from Applied Biosystems, Madrid, Spain) in a final volume of 20 *μ*L containing 10 *μ*L of Power SYBR Green RT-PCR Mix (2X), 1 *μ*L of 10 *μ*M primer forward, 1 *μ*L of 10 *μ*M primer reverse, 0.16 *μ*L of RT Enzyme Mix (125X), 1 *μ*L of RNA (100 ng/*μ*L), and 6.8 *μ*L of RNase-free water. Forward and reverse primers for NEP and Ubi were designed specifically for qPCR (Table 2). The qPCR reactions were performed in an ABI 7300 Real-Time PCR System (Applied Biosystems) using 96-well plates in triplicate with no-template controls as negative controls in all plates. The following thermal cycling conditions were used: (1) reverse transcription at 48°C for 30 min, (2) activation of AmpliTaq Gold DNA Polymerase at 95°C for 10 min, and (3) amplification for forty cycles at 95°C for 15 sec and at 60°C for 1 min. Finally, a melting curve analysis confirmed the amplification specificity. Relative quantification was analyzed by direct comparison between the cycle threshold (Ct) values of NEP and Ubi (this latter is the house-keeping gene) of the samples, using the comparative 2^−ΔΔCt^ method [25]. Expression of NEP is presented as relative units (ru), which are the values of 2^−ΔΔCt^.

### 2.5. Quantification of Cholinergic Neurons in Basal Telencephalon and Extent of A*β* Deposits in Gyrus Proreus

Brains of dogs included in this study were analyzed previously for quantification of cholinergic neurons in basal telencephalon and extent of A*β* burden in *gyrus proreus *by Insua et al. [17]. This work was carried out following stringent stereological procedures, as previously described; in brief, brains were removed immediately after death and processed for immunohistochemistry with an anti-p75NTR monoclonal antibody (ME20.4, Sigma). In addition, a block containing the gyrus proreus (prefrontal cortex) was prepared to evaluate A*β* deposits by immunohistochemistry with monoclonal antibody 6E10 (from Covance). Labeled neurons and A*β* deposits were analyzed using a microscope with a stereological system (StereoInvestigator software from MicroBrightfield, Germany). The optical fractionator method was used to estimate the total number of p75NTR positive neurons in one out of 20 forebrain sections by an operator blinded to the animal's diagnostic classification. The extent of A*β* deposits labeled with the 6E10 antibody was estimated by the area-fraction fractionator (StereoInvestigator) in one section of gyrus proreus per dog.

### 2.6. Statistical Analysis

The NEP expression data obtained by qPCR were statistically analyzed with PASW 18.0 software (SPSS Inc.). NEP expression levels were compared between the three groups of dogs by a nonparametric Mann-Whitney test. The null hypothesis was rejected if *P* < 0.05. Correlation between NEP expression and brain amyloid deposits or number of cholinergic neurons was assessed (Spearman test) using previously published data from the same cohort [17].

## 3. Results 

### 3.1. Comparative and Semiquantitative mRNA Analysis of NEP and IDE in the Brain of Healthy and Demented Dogs

**Figure 1:**
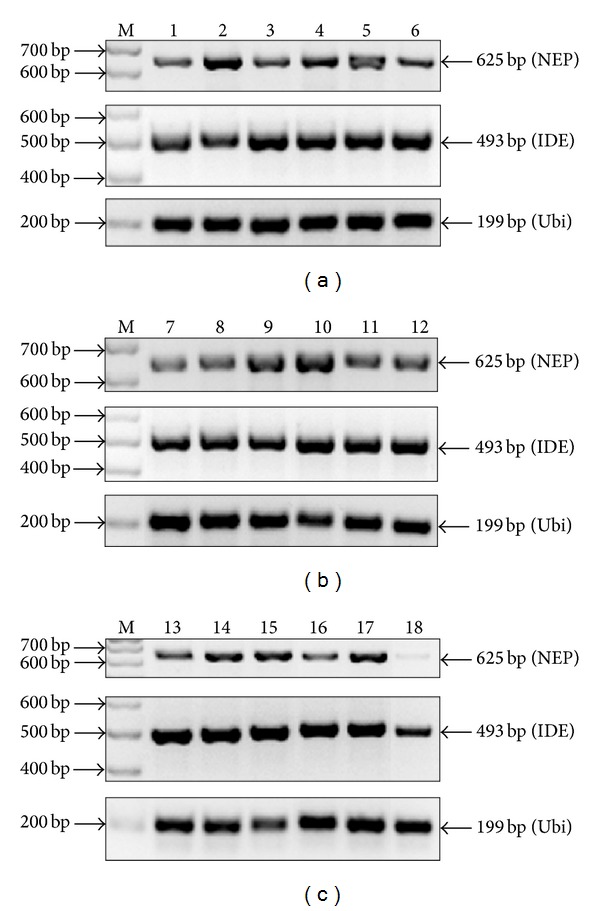
Agarose gel electrophoresis of RT-PCR of NEP, IDE, and Ubi arranged by groups ((a) young, (b) aged-CU and (c) aged-CI). Amplicons of NEP cDNA (625 bp band) in young (a) and aged-CU (b) animals showed considerable intragroup variability; in contrast in aged-CI dogs (c), there are lower levels of NEP with less variability. IDE amplicons (493 bp band) present high intensity of band with similar levels in all animals. Ubi was included as an internal standard.

Dog brain total RNAs were processed by RT-PCR with posterior analysis in agarose gel electrophoresis. Amplicons of NEP (exons 4 to 9, primers in Table 2) and IDE (exons 15 to 19, primers in Table 2) cDNAs (with Ubi as internal standard) are visualized in Figures 1(a)–1(c). Substantial differences between the expression pattern of NEP and IDE mRNAs were found. Whereas IDE mRNA was homogenously expressed in all the animals at similar levels than Ubi, NEP mRNA expression varied in both intragroup and intergroups of animals. The intragroup variability of NEP mRNA was particularly notorious in the young and aged-CU groups. By contrast, the electrophoresis bands of NEP mRNA in the aged-CI dogs appeared more homogeneous and less intense than in the other two groups.

### 3.2. Quantification of Brain NEP mRNA Expression in Healthy (Young and Aged) and Demented Dogs by Real-Time PCR

Results of relative quantification of NEP mRNA expression, by qPCR, were congruent with the previous semiquantitative analysis (Table 1). The main result was the significant difference (*P* < 0.01) in the brain NEP mRNA expression levels between aged-CU and aged-CI groups. On average, NEP mRNA expression was 5 times higher in the aged-CU than in the aged-CI dogs (mean ± S.D.: 2.25 ru ± 1.35 versus 0.45 ru ± 0.23, resp.). This difference was also significant (*P* < 0.01) when healthy animals (young and aged-CU) were pooled together and compared with demented animals (2.2 ± 1.48 versus 0.45 ru ± 0.23, resp.). In contrast, no significant differences existed between young and aged-CU dogs (2.15 ru ± 1.72 versus 2.25 ru ± 1.35, resp.).

**Figure 2:**
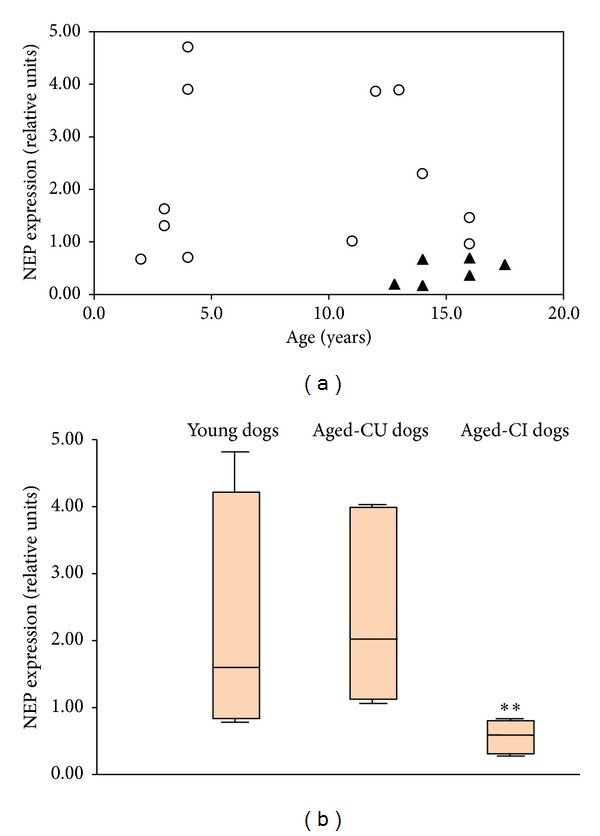
Diagrammatic representation of dog NEP expression. (a) Relative quantification of brain NEP expression in dogs (cognitively healthy (o), both young and aged, and aged-cognitively impaired (▲)) is presented as a function of age. (b) The box-and-whisker plot showed a larger dispersion in young and aged-CU dogs than in the aged-CI dogs. ** indicates *P* < 0.01 with regard to both, aged-CU and young dogs.

Interestingly, the intragroup variability of NEP mRNA expression was greater in the young and aged-CU animals than in the aged-CI group (Figures 2(a) and 2(b)). Thus, the range of measurements within the young group was between 0.67 and 4.71 ru (interquartile range 3.41 ru) and in aged-CU dogs was between 0.96 and 3.89 ru (interquartile range 2.87 ru) whereas the aged-CI group ranged from 0.17 to 0.7 ru (interquartile range 0.48 ru) (Table 1 and Figure 2(b)).

### 3.3. Analysis of Correlation between Cortical NEP mRNA Expression and Both the Number of Cholinergic Neurons in Basal Encephalon and the Extent of A*β* Deposits in Gyrus Proreus

**Figure 3:**
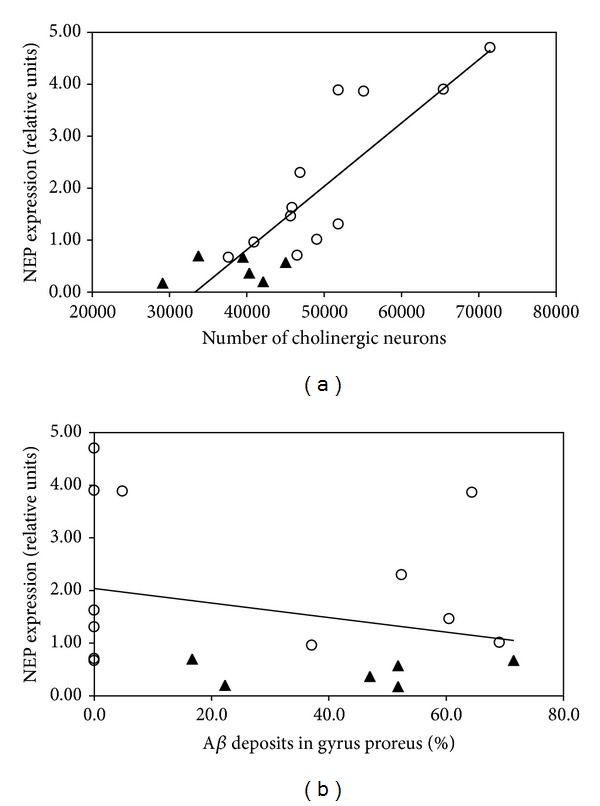
Statistical analysis of the correlation between cortical NEP mRNA expression and the number of cholinergic neurons in basal encephalon (a) and the extent of A*β* deposits in gyrus proreus (b). A positive correlation exists between the number of cholinergic neurons of basal brain with the prefrontal NEP expression (*R* ^2^ = 0.73), while no correlation is seen between NEP expression and insoluble A*β* deposits in the same prefrontal cortex (in this diagram, dogs without A*β* deposits belong to the young group). Cognitively healthy dogs are indicated with (o) and aged-cognitively impaired dogs with (▲).

The total number of basal forebrain cholinergic neurons in the brain of these animals had been previously assessed by unbiased stereological procedures [17]. We used those previously published data to explore the possible association between the number of basal forebrain cholinergic neurons and the level of NEP mRNA expression in frontal cortex because these two processes have been related to cognitive impairment in CDS and AD. It resulted that this correlation was significant for the whole study population (*P* < 0.001; *r* = 0.853) (Figure 3(a)); statistical significance was maintained when only the group of elder animals (healthy and demented) was taken into account (*P* < 0.01; *r* = 0.797).

In contrast, no correlation was found between the levels of NEP mRNA expression and deposited insoluble A*β* burden in the prefrontal cortex of these animals (Figure 3(b)). Quantification of A*β* burden, as percentage of occupied area, had been previously quantified [17].

## 4. Discussion

In the present study we have sought differences of expression of the most important A*β* peptidases (NEP and IDE) between healthy and CDS-affected dogs. In an initial semiquantitative RT-PCR we determined that the expression of IDE was not substantially modified in demented dogs, in comparison with their aged-CU partners. Nevertheless, NEP presented a more variable expression, and we analyzed, more precisely by qPCR, whether NEP mRNA expression levels were related to aging and/or cognitive status in dogs. We assumed that levels of mRNA NEP are indicative of protein amount and enzymatic activity, as has been reported in other studies [26].

Our results showed a significantly lower expression of NEP mRNA in aged-CI dogs than in their aged-CU counterparts. Similar results have been obtained in humans, where AD brains presented 20% lower NEP mRNA expression than control brains [8]. All brains of the aged-CI dogs presented low levels of NEP expression, suggesting that high NEP expression levels might play a protective role against canine CDS. Furthermore, it is also possible that low NEP expression could be a risk factor for developing CDS at advanced ages. However this is only a possibility and further experiments are necessary to ascertain it. Nevertheless, our results do not support the idea that low brain NEP expression alone could be sufficient for the development of CDS, because some of our aged-CU dogs also displayed low levels of NEP.

On the other hand, levels of NEP mRNA expression in the young and aged-CU groups presented an elevated variability among individuals, with high and low levels of NEP; nevertheless the values of mean and standard deviation are similar between both groups. This result suggests that age might not be a relevant factor for modulating the expression of NEP gene in dogs. In this sense, contradictory reports have been published regarding the possible variation of NEP expression levels with relation to age. Whereas Miners et al. [12] did not find significant differences in the NEP protein levels in frontal cortex of humans ranging from 16 to 95 years, other authors found an age-dependent decline of brain NEP in humans and mice [10, 11]. Further experiments are necessary to elucidate this.

As we have indicated above, CDS is an experimental model for AD; two features of these processes are a gradual impairment of the cholinergic system and the appearance of A*β* deposits in cortical areas. Nevertheless, until now, it has not been possible to establish a direct relation between both phenomena. Our statistical analyses showed a strong positive correlation between the level of NEP mRNA expression in the frontal cortex and the number of basal forebrain cholinergic neurons, previously reported for this same cohort of dogs [17]. Although these basal forebrain cholinergic neurons do not express NEP, low expression of NEP in the cortical area could lead to high levels of toxic soluble A*β* isoforms that might eventually affect cholinergic neurons through their axonal projections to the cortex or simply by diffusion of the toxic A*β* species to the basal forebrain [27, 28]. On the other hand, no significant correlation was found between NEP mRNA expression and the extension of insoluble A*β* deposits in the canine frontal cortex. Similar results were obtained by Miners et al. [12] in controls and AD human brains, where no correlation was detected between NEP protein levels and insoluble A*β* in frontal cortex, a circumstance that permits us to speculate if NEP presents greater affinity for oligomeric and soluble forms of A*β* (currently accepted as the main neurotoxic species [29, 30]) than for insoluble A*β* deposits.

In conclusion, canine CDS is presented as a useful experimental model for AD, which could be relevant to the development of screening tools for determining risk within a given population in order to apply therapeutic strategies for A*β*-related neurodegenerative diseases, in which NEP appears as a key peptidase.

## Figures and Tables

**Table 1:** Characteristics and NEP expression values of the animals.

Dogs	Breed	Sex	Cause of death	Age (years)	NEP expression (ru)
Young (2–4 years)					
1	Mongrel	F	Babesiosis	4.0	0.706
2	Beagle	M	Hunting injuries	4.0	4.705
3	Epagneul Breton	M	Hunting injuries	2.0	0.669
4	Ibizan Hound	M	Urethral rupture	3.0	1.308
5	Mongrel	M	Car accident	4.0	3.902
6	Mongrel	M	Rodenticide toxicosis	3.0	1.627
			Mean ± S.D.	**3.3 ± 0.8**	**2.15 ± 1.72**

Aged-CU (11–16 years)					
7	Mongrel	M	Urolithiasis	16.0	0.961
8	Mongrel	F	Mammary tumors	14.0	2.298
9	Teckel	F	Intervertebral disk disease	13.0	3.888
10	Fox Terrier	M	Chronic kidney disease	12.0	3.864
11	Mongrel	M	Tracheal collapse, bronchitis	11.0	1.014
12	Toy poodle	M	Mitral valve disease	16.0	1.462
			Mean ± S.D.	**13.7 ± 2.1**	**2.25 ± 1.35**

Aged-CI (12–17 years)					
13	Chiwawa	M	Severe cognitive damage	17.5	0.571
14	Mongrel	M	Severe cognitive damage	16.0	0.366
15	Cocker Spaniel	M	Severe cognitive damage	12.8	0.199
16	Toy poodle	F	Severe cognitive damage	14.0	0.671
17	Pekingese	M	Severe cognitive damage	16.0	0.696
18	West Highland	F	Severe cognitive damage	14.0	0.173
			Mean ± S.D.	**15.05 ± 1.7**	**0.45 ± 0.23**

**Table 2:** Pairs of primers used for PCRs.

Amplified gene	Primer names	5′→3′ sequences	Annealing temperature (°C)	Product length (bp)	GenBank accession number
Dog NEP (exons 4 to 9)	NEP1U20 NEP625L20	F: ATGGGCAGATCAGAAAGTCA R: TATCATCCGTGCCAACAAAA	57	625	JF451103

Dog IDE (exons 15 to 19)	IDE1815U20 IDE2307L20	F: CCTCAAAGACTCACTCAACG R: CAGCTGACTTGGAAGGAGAG	53	493	XM534963

Dog Ubi	Ubi145U20 Ubi343L20	F: CAGCTAGAAGATGGCCGAAC R: ACTTCTTCTTGCGGCAGTTG	53	199	AB032025

Dog NEP (for qPCR)	NEP1575U20 NEP1728L20	F: CCGAGAAAAAGTGGACAAGG R: ACCCCCGTAGTTCAAGGAGT	60	154	JF451103 JF713705

Dog Ubi (for qPCR)	Ubi190U20 Ubi344L20	F: GAGTCCACCTTGCACTTGGT R: CACTTCTTCTTGCGGCAGTT	60	155	AB032025
